# Changes in the forest ecosystems in areas impacted by aridization in south-western Romania

**DOI:** 10.1186/2052-336X-12-2

**Published:** 2014-01-06

**Authors:** Remus Pravalie, Igor Sîrodoev, Daniel Peptenatu

**Affiliations:** 1 Faculty of Geography, Bucharest University, Bucharest, Romania; 2 Moldavian Academy of Sciences, Institute of Ecology and Geography, Chisinau, Republic of Moldova; 3 Interdisciplinary Center for Advanced Research on Territorial Dynamics, University of Bucharest, Bucharest, Romania

**Keywords:** Degradation, Deforestation, Aridity, South-Western Romania

## Abstract

**Background:**

In the past few decades, global climate change has accentuated the intensification of aridization in South-Western Romania, with direct and indirect consequences on the quality of forest ecosystems. In addition to qualitative deterioration, the quantitative changes brought about by intensive anthropic deforestation have created the conditions for a decline in the size of forest areas on vast tracts of land. The paper aims to analyze the qualitative and quantitative changes in the forest ecosystems in South-Western Romania, changes due to the synergic context of the global climate changes and the anthropic pressures of the past three decades. In order to capture the evolution of aridization in the study area, specific aridization indexes have been calculated, such as the De Martonne index and the UNEP aridity index. 1990 and 2011 satellite images have been used in order to quantify the qualitative changes.

**Results:**

The results obtained indicated that, in the past two decades, the quality of the biomass declined as a result of the increase in the climatic aridity conditions (De Martonne si UNEP aridity index, indicating in the last decades, annual values under 15 mm/°C, and under 0.5 mm/mm, that means that the values situated under these thresholds, describe arid and semi-arid climate conditions). Also, the uncontrolled logging across vast surfaces caused the loss of forest ecosystems by 7% in the overall study area, during the last three decades.

**Conclusions:**

The severe effects of aridization meant, first of all, a significant decline in the quality of the ecosystem services supplied by forests. In the absence of viable actions to correct the present situation, the extremely undesirable consequences of an ecological and social nature will arise in the near future.

## Introduction

The increasingly strong climate changes of the past few decades have brought about important changes in the forest ecosystem worldwide [1-4]. Drought, one of the most important consequences of this phenomenon, has caused changes at the level of forest ecosystems, such as a decline in bioproductivity, changes at the level of the structure of ecosystems, an increase in the fragmentation of forest landscapes [5], as well as changes at the level of the physiological processes [6,7]. The survival of the forest ecosystems in the context of environment changes depends mainly on their adaptation potential [8], but the gross variations of the climate variations may profoundly endanger that capacity [9].

According to the latest IPCC report (2007) [10], Romania is one of the top seven countries in Europe in terms of aridity (desertification) risks. Recent studies confirm the steep aridization trends in certain regions of Romania [11] with direct consequences on both the deterioration of the land and on the deterioration of the forest systems [12]. Among the most important changes inside the forest ecosystems one must remark those of a biocenotic nature such as the phenology, the productivity, the drying up of forest vegetation species, obstacles against the natural regeneration of the forests, among others [13].

Our study focuses on quantification of the changes that occurred inside the forest ecosystems in a region of Romania that faces the severe effects of aridization, Southern Oltenia. The transformations of the forest ecosystems in the region analyzed have been caused by two distinct processes: degradation and logging. Degradation involves the loss of the forest’s initial characteristics because of certain factors (climate stress, pollution etc.), so that the forest can no longer offer quality ecosystem services. Forest logging is accompanied by replacing forest-covered areas by land with some other destination [14]. The analysis of the spatial and temporal dynamics of the forest ecosystems is necessary for a sustainable administration of those ecosystems, especially in the context where the escalation of the fragmentation and degradation processes may cause perturbations at the level of the geographical landscapes [15].

**Figure 1:**
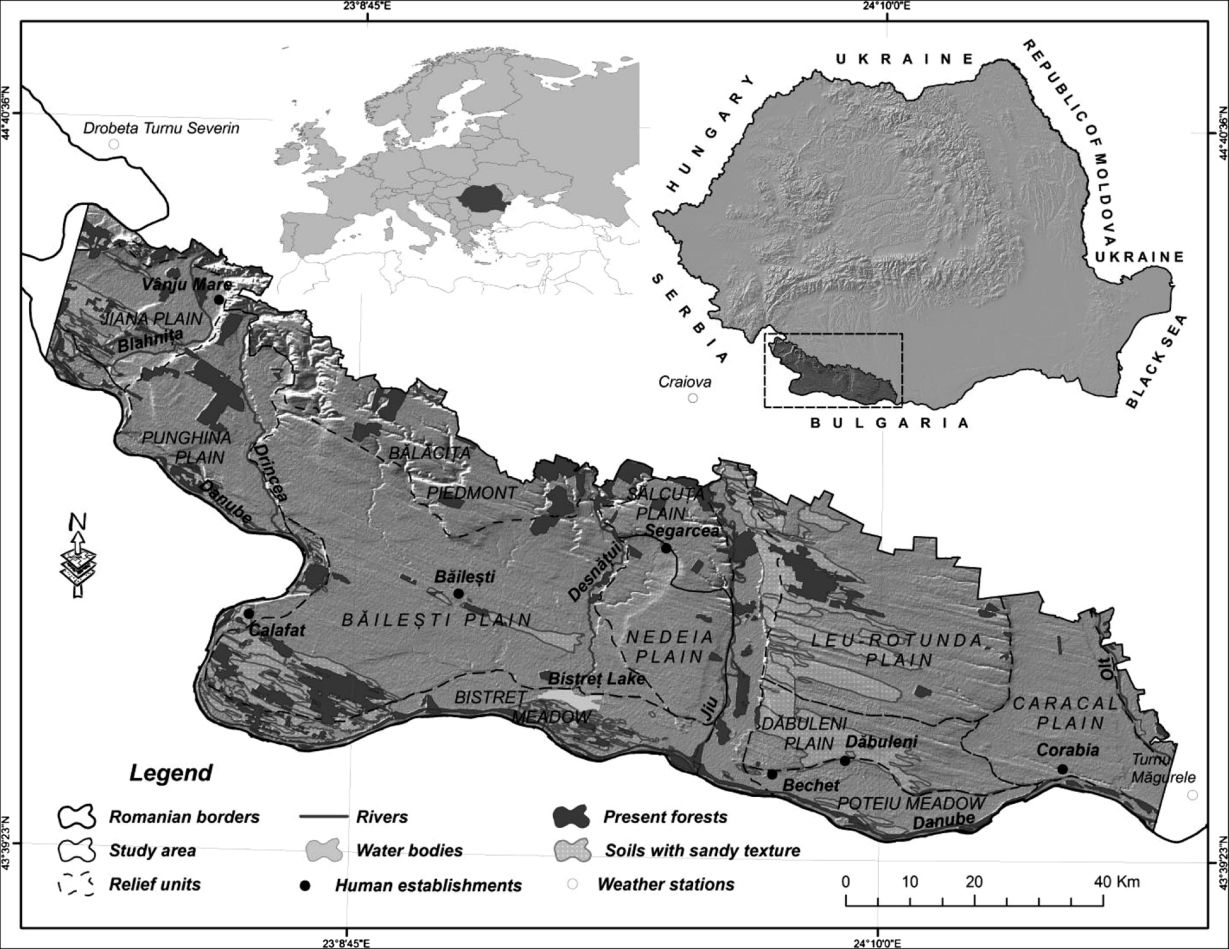
Location of the study area, Southern Oltenia, in Romania.

The study area consists in the lowlands in South-Western Romania (Southern Oltenia) (Figure 1). The administrative criteria delineate the study area, and it contains 113 territorial administrative units, in 3 departments (Mehedinti, Dolj and Olt). From the geographical point of view, its limits are defined by the Danube valley and the Romanian border (the Southern border), the Getic Plateau (the Northwest border), the Romanian Plain (northeast border) and by the availability of the satellite images (Eastern and Western borders).

The relief is conducted on an amplitude of 200 m (25 m in the Danube floodplain and the maximum values of 240 m in the area of the Getic Plateau), with consequences for spatial potential differentiation of the ecological forest ecosystems. This distinction lies in richest precipitation (up to 640 mm/year) and stress lower heat decreased (below 11°C), in the area of the Getic Plateau, compared with the south, where rainfall decreases to 563 mm/year and the average annual temperature rises above 11.5°C [16], the thermal threshold is generally considered the critical threshold triggering drying acacia forest species (the main forest species present in the area of study) [17-21].

Another feature of the landscape is related to the presence of sand dunes, east of the Danube and Jiu, these deposits reaching even up to 20 m thick in plains areas Dabuleni and Leu-Rotunda [22] (Figure 1). Related to the presence of these deposits is the existence of soils with sandy texture, consisting principally of chernozem cambic, generally found in the area of River and the Danube, and psamosoil, extended over large areas, especially in the eastern part of Jiu River [23] (Figure 1). These soils represent a limiting factor for the body of woods, especially in the summer season when, due to days with frequent high temperatures (above 30°C), in the context of the advection of warm air from North Africa, there is a heating surface layer of sand approx. 60–70°C, affecting especially young stands [17-21]. Textured sandy soils may be a limiting factor in the optimal development of biotic layer both by the very low content of organic elements, and also very low retention capacity of soil water reserves, in the context of high permeability.

The predominant forest vegetation in the study area consists in mixed-vegetation lowland woods of mainly downy oak (*Q. pubescens*), Turkey oak (*Q. cerris*), Hungarian oak (*Q. frainetto*) species, as well as pedunculate oak (*Q. pedunculiflora*), Tatar maple (*Acer tataricum*), field maple (*Acer campestre*) species, among others, on isolated areas [24]. Azonal forest vegetation such as false-acacia (*Robinia pseudoacacia*) forests, forests found on the sandy deposits in the Western parts of the Dabuleni and Leu – Rotunda plains, the South-West of the Bailesti plain (Rast cape) and the central-Western part of the Jiana plain (Figure 1) are of particular interest to our study.

In the past century, the study area underwent important changes in the spatial distribution and the flora composition of the forests, in two main phases. During the first half of the 20^th^ century there was large-scale afforestation, especially false-acacia, in the form of shelter belts, so as to stabilize sand dunes, whose expansion threatened inhabited areas [25,26]. In addition, false-acacia forests were of vital importance to lowering the negative effects of deflation, fighting soil degradation and preserving an environmental balance of the soil [27,28]. Canadian poplar (*Populus canadensis*) is yet another hybrid species, artificially brought in, especially for ecological purposes, more precisely as a replacement of indigenous little productive species, such as silver poplar (*Populus alba*) and black poplar (*Populus nigra*).

In the second half of the 20^th^ century the reverse process, destruction of these areas with protective destination, gains in scale [29,30] in favor of agricultural land. For instance, 12,000 hectares of forest land were cleared in 1969 [29]. This process led to the reactivation of the sand dunes. As a result, there was a return to stabilizing the sand dunes by means of afforestation during 1970–1974 and in 1979 (1,600 ha). Later on, law 18/1990, concerning land distribution, led to the escalation of the clearing of both the forests and the shelter belts, as well as to the fragmentation of the forest ecosystems, among others.

In addition to the socio-economic factors, the natural factors, too, impacted on the quality and productivity of forest vegetation in the past few decades. Nowadays, the study area is midway through a full-scale aridization, and specialized studies at the regional level confirmed this phenomenon by means of climate change [31-35], landscape dynamics [29], by highlighting the dynamic of forest areas with the direct effects of the activation of the extant sand-dunes in the region [30,36,37], among others. The rise of the temperature and the drop in the average annual precipitations of the past three decades have brought about, in many situations, the drying up of the vegetal layer and of the very forest species [29,35].

Around 16% (116,300 ha) out of the total study area (736,723 ha) is covered by sandy-texture soil overlapping sand deposits [23]. These deposits, coming in from the Danube and Jiu river valleys by means of the wind erosion process, require a constant work of covering them up with forest vegetation so as to fight wind action. In the past two decades, a large part of the sand deposits have been reactivated as a result of the deficient administration of the forest ecosystems at the local level (illegal clear-cutting, the local authorities’ deficient policies in the administration and protection of the forest fund, among others) and at the national level (the absence of viable strategies to fight the ensuing stabilization).

Nowadays, climate changes, combined with other local factors such as the improper use of the tracts of land, especially after 1990, the year of political transition (clear-cutting, destruction of the vineyards, the abandonment of irrigation systems, with direct effects of the water layer levels, the abandonment of the rice crops with a key role in preserving humidity, among others) have created the premises of the escalation of the aridization process in Southern Oltenia with direct consequences on the forest ecosystems. Thus, three fundamental components of the environment were altered: the destabilization of the sand deposits found on large areas in the region analyzed, the changes of the local climate and the changes in the destination of the tracts of land, with the direct consequences of an ecologic, climatic and socio-economic nature.

## Methods

A first phase consisted in the analysis of the evolution of the aridization process in the study area. This analysis was conducted by means of two indexes: the De Martonne index and the UNEP aridity index. The time span analyzed was 50 years (1961–2009), and the weather data (temperature and precipitations) of the local Drobeta Turnu Severin, Craiova and Turnu Magurele weather stations come from the European climate database ECAD (European Climate Assessment & Dataset) [38].

The gaps in the data series from the Drobeta Turnu Severin and Turnu Magurele weather stations were filled in by means of a linear regression by means of the data from the Craiova weather stations, the sole weather station in the area with an uninterrupted set of data. Because of the proximity and to a relative similarity of the physico-geographical contexts of the three weather stations, the correlation between the data series was a close one (the *r* correlation quotient exceeded 0.92), justifying the use of the respective method.

The De Martonne aridity index (I ar-DM) was calculated on the basis of the connection between temperature and precipitations [39]: I ar-DM = P/(T + 10), where P and T are precipitation (mm), and average annual temperatures (°C), respectively. The UNEP aridity index (Iar-P/PET), suggested by the United Nations Environment Programme in 1992, was calculated on the basis of the formula: Iar-P/PET = P/PET [40], where P means average annual precipitations (mm), and PET is potential evapotranspiration (mm) calculated by means of the Thornthwaite methodology [41], the methodology most frequently used in Romania’s case [42].

Although these aridity indexes can be calculated rapidly, not factoring in many climate variables, they are genuinely useful, because they offer a big picture of the climate changes and therefore of the changes in the ecologic potential at the local level, with direct consequences on the quality of forest ecosystems in the region.

The second phase of the analysis consists in quantifying the effects of the *degradation* and *logging* processes on the forest ecosystems in the study area. Processing and using satellite images is an efficient manner of identifying natural perturbations (such as climate stress) that occur at the level of forest ecosystems [43]. In the case of the present study, too, the use of satellite images and processing digital indexes are a satisfying way of assessing the deterioration of the state of the forest ecosystems, especially in the context where those ecosystems are in one of the regions worst hit by aridization in Romania.

In assessing the degradation of forested areas, satellite images were used with a view to obtaining specific digital indexes in the analysis of the analysis of the quality of the biomass: NDVI and MSAVI 2. We selected the year 1990 and 2011 in order to cover as closer as possible the economic transition period in Romania (which started after the Revolution in December 1989). Confining our analysis to the transition period was deliberate, because in the study area not just natural trends have been changing in the past two decades, but socio-economic characteristics (including agriculture and land-use patterns) have changed as well, and 1990 represents the threshold year. Our choice took also into consideration the availability of the images, as well as a relative coincidence of the day of acquiring the scenes (11 July 1990 [44] and 22 August 2011 [45]) in order to minimize likely differences in phenological state of the forest vegetation. Image processing steps followed standard procedure of radiometric calibration and atmospheric correction of Landsat 5TM images, used by U.S. Geological Survey [46,47]. There were two scenes for each time-slice, located on the same path (184) and two neighboring rows (029 and 030); with about 1 min time difference in acquiring the scenes within each pair we can consider that no temporal shift correction is needed. Taking account that our study area represents a plain, with quite low values of relief energy, it was considered that a topographic correction is not necessary in this case.

The NDVI index is particularly important in the analysis of the quality and the productivity of the biomass [48], and it is therefore very useful in the analysis of the degradation of forested areas*.* It features the major benefits of being relatively easy to calculate and of supplying qualitative information on the state of the vegetal layer on large surfaces:

$$\mathrm{NDVI}=\frac{\left(\mathrm{NIR}–\mathrm{RED}\right)}{\left(\mathrm{NIR}+\mathrm{RED}\right)},$$

where NIR – near infrared spectral band, RED – red spectral band of a multispectral satellite image. This index was successfully used in various studies on the degradation of the forest ecosystems in various areas of the world suffering from aridization/desertification [49-51].

The theoretical values of the NDVI index range from -1 to + 1, with the minimal values demarcating areas with no vegetation, and the maximal values demarcating areas with high-density vegetation. In fact, the extreme values are never reached, in general the real span of the index ranges from around -0.8 to + 0.9. The areas without vegetation, with exposed rock, sand or water are assigned values below 0.1 while high-density vegetation is assigned values above 0.3.

The MSAVI2 index is another means to assess the degradation of forested areas, coming in to supplement the NDVI index by measuring the degree of brightness of exposed soil. Although it was initially developed as the SAVI (Soil Adjusted Vegetation Index) [52] and then modified as the MSAVI2 index [53], the index proved to be quite efficient in specialized analysis work, and it was later borrowed in various studies [54-57].

It is calculated as follows: $\mathrm{MSAVI}2=\frac{2\mathrm{NIR}+1-\sqrt{{\left(2\mathrm{NIR}+1\right)}^{2}-8\left(\mathrm{NIR}-\mathrm{RED}\right)}}{2}$, and value fluctuation is similar to that of the NDVI index (values below 0.1 are typical of exposed soil, sand, exposed rock or bodies of water).

The data used to assess the effects of forest clear-cutting come from two distinct sources which span an interval of 25 years: 1:25,000-scale topographic maps, the 1981 edition, and the Corine Land Cover European database for 2006.

The analysis of the evolution of the forested areas was supplemented by three case studies. Those case studies amount to circa 16% of the total surface of the study area (116 thousand hectares out of 737 thousand hectares), and they are mostly located in the region of sandy soils and sand dunes, an area characterized by one of the most vulnerable landscapes in Romania.

In the case of two out of the three examples, an analysis of the evolution of the forest species was conducted by means of the method of the Markov land use transition matrix, a method frequently used in the analysis of spatial and temporal changes in land use [58,59]. The data regarding the distribution of the dominant forest species in the past was extracted from the topographic maps (the 1981 edition). The data on the present distribution of the species come from two sources: the contours of the areas they cover were extracted from 1:5,000-scale orthophotographic plans (the 2008 edition), and the information on the floral composition of those areas was extracted from the latest forestry-planning documents (Forestry planning documents of the Jiana (1997 [17], Sadova (2003 [18], Calafat (2004 [19], Poiana Mare (2004) [20] and Simian (2010) [21] forestry districts). The selection of the areas where the respective method was used was determined by the availability of the sources of information.

Eventually, a cluster analysis was conducted at the level of territorial-administrative units, starting from two indicators: the total surface of the forests and the increase/decrease (in absolute units) of the forested areas within the borders of the respective territorial-administrative units. Clusterization was achieved by means of the R statistical programming language by means of Ward’s method, using the Euclidian distance.

## Results

### Climate aridization

**Figure 2:**
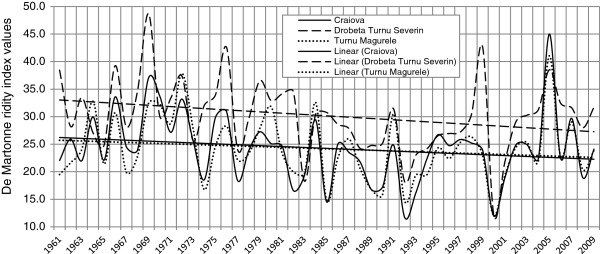
Annual evolution of the De Martonne aridity index and the linear trend at the Craiova, Drobeta Turnu Severin and Turnu Magurele weather stations (1961–2009) (data processing ECAD).

The De Martonne aridity index features a major fluctuation during the time period analyzed, with its values on a downward trend (Figure 2). The lower the values, the more excessive the aridization [39]. The most visible downward trend of the De Martonne index values is registered after 1982 when the values start to fluctuate within the 15–20 range (mm/°C), a range that, according to the De Martonne classification, is typical of arid steppe areas.

**Figure 3:**
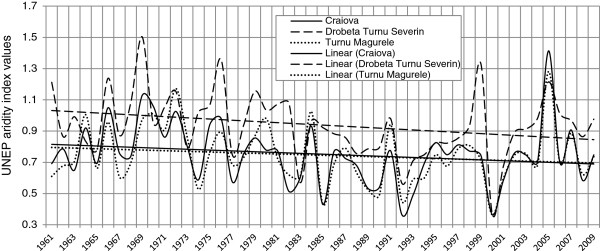
Annual evolution of the UNEP aridity index and the linear trend at the Craiova, Drobeta Turnu Severin and Turnu Magurele weather stations (1961–2009) (data processing ECAD).

In turn, the UNEP aridity index values also display a downward trend (Figure 3). In their case, too, the lower the values, the more arid the climate. Starting 1985, the index values frequently fall in the 0.2 - 0.5 range (mm/mm), a range typical for a semiarid climate [40].

### Forest ecosystem degradation

**Figure 4:**
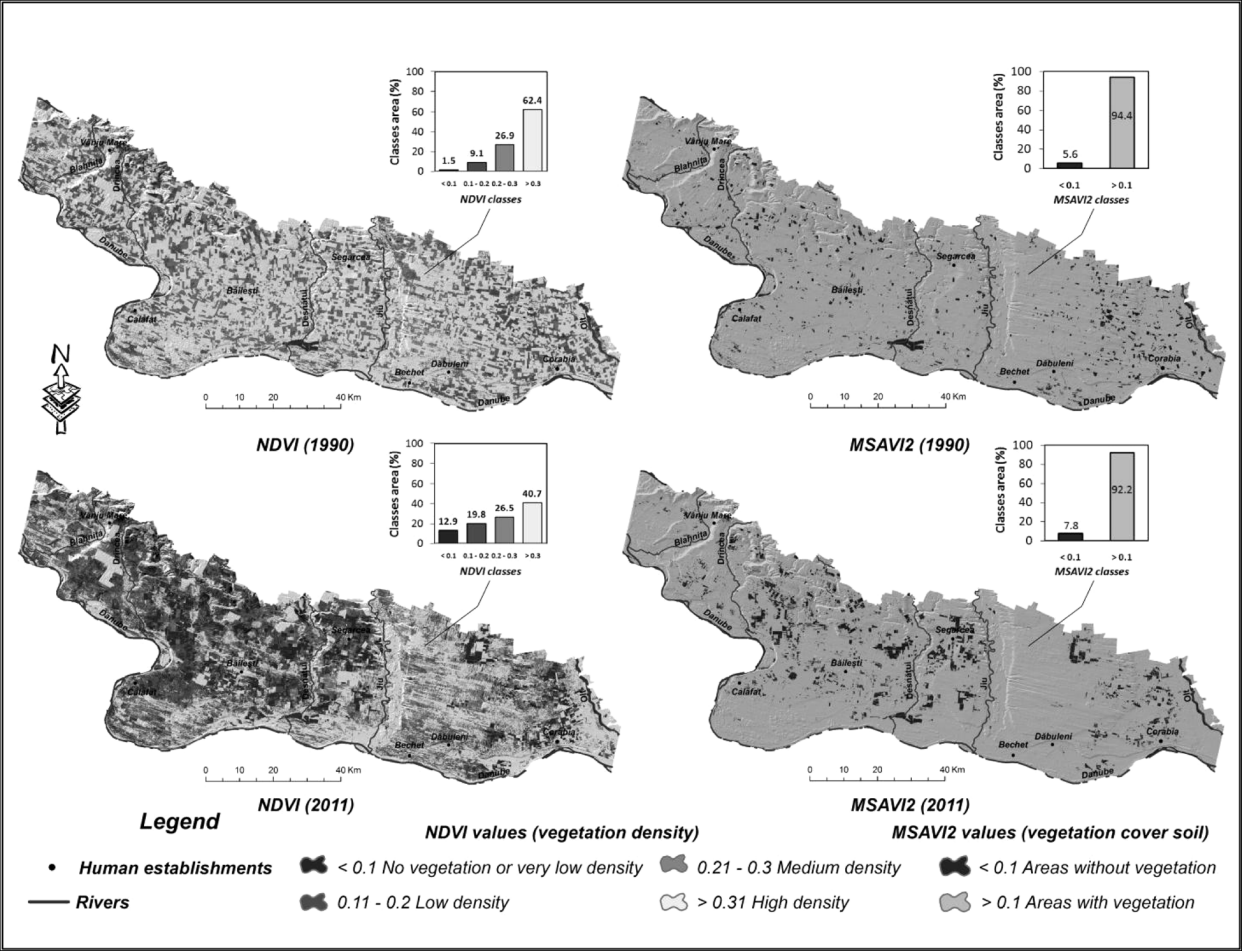
Spatiotemporal evolution of the NDVI and MSAVI2 index values from 1990 to 2011.

The two indexes analyzed (NDVI and MSAVI2) allow a visual and quantitative assessment of the changes in the quality of the vegetation. The spatiotemporal analysis of the NDVI index in the two reference years (Figure 4) offers an overall view of the degradation of the vegetal layer. Because the 0.1 – 0.2 and 0.2 - 0.3 index groups are not typical of forest ecosystems, but merely areas covered with more-or-less-developed vegetation, the comparative analysis focused on the value > 0.3 group. In general, values > 0.3 match high vegetation density [60] and therefore, to a certain extent, forest ecosystems.

While by 1990 the areas matching the > 0.3 value group covered an area of around 459,460 hectares (62%) of the total area, by 2011 they only amounted to 299,450 hectares (40%). Thus, over the 20-year time span analyzed, the areas with high-density vegetation shrank by 20%.

Indirectly, the analysis of the changes occurring at the level of vegetation-less areas, matching the value < 0.1 group, offers information on the degradation of the vegetal layer. Over the time span analyzed, exposed-soil areas had expanded by 88% by 2011 (95,382 hectares) compared to 1990 (11,052 hectares), a situation mainly due to climate-related causes.

The MSAVI2 index, indirectly, provides yet another opportunity to assess the evolution of the ecological state of the vegetal layer by means of the analysis of the evolution of exposed areas, areas lacking any vegetation. The steep expansion of the exposed areas (< 0.1) in the past two decades (Figure 4), highlights important changes in vegetation, as in its disappearance. Thus, while by 1990 exposed areas covered a surface of around 38,900 hectares (5.6% of the total surface), by 2011 they covered around 53,600 hectares (close to 8% of the total).

**Figure 5:**
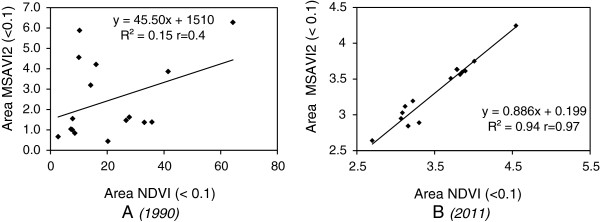
**Spatial correlation (R**^**2**^** coefficient of determination, r – correlation coefficient) across relief units of the values matching exposed areas (< 0.1) of the NDVI and MSAVI2 index by 1990 (panel A) and 2011 (panel B).**

The analysis of the exposed areas, classified below the 0.1 threshold, reveals a similar spatial evolution in the case of the NDVI and MSAVI2 indexes in the two reference years. By matching these threshold values against the 16 relief units present in the analyzed area, one notices a similar expansion of the areas they cover. Thus, significant increases in the critical areas occurred in the central region (the Bailesti, Nedeia and Salcuta plains) and the North-Eastern region (the Leu-Rotunda and Caracal plains) of the study area. The correlation of the spatial evolution of the exposed areas, obtained by means of linear regression (Figure 5), indicates a close connection in the case of the 2011 reference year.

**Figure 6:**
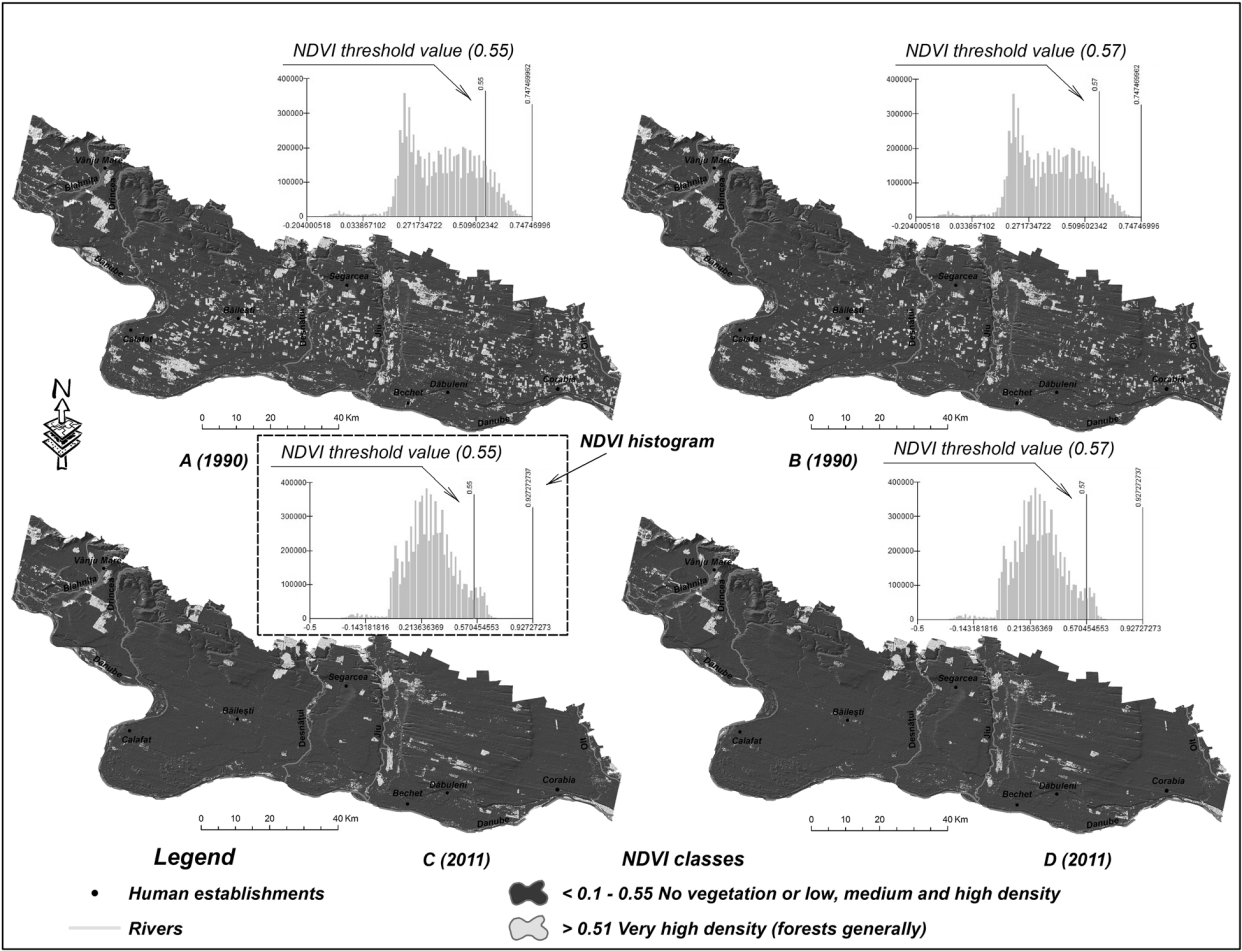
**Spatiotemporal evolution of the areas with very-high-density vegetation (in general forest areas) matching the upper limits of the NDVI index between 1990 and 2011:** Panel **A** Very-high-density vegetation in 1990 with NDVI threshold value 0.55; Panel **B** Very-high-density vegetation in 1990 with NDVI threshold value 0.57; Panel **C** Very-high-density vegetation in 2011 with NDVI threshold value 0.55; Panel **D** Very-high-density vegetation in 2011 with NDVI threshold value 0.57.

In order to take into consideration the qualitative changes of the forest ecosystems and not of the vegetal layer in all, the NDVI index is particularly useful in delimiting the higher thresholds of the respective values. In general, the 0.6 threshold is typical of forest areas in temperate regions [60], but, in the case of the study area, this threshold lowers the very-high density areas (typical of forest areas) too much, so that a proper threshold would be one roughly included in the 0.55 – 0.57 range (Figure 6).

By setting the threshold of the NDVI index in the two reference years at 0.55 one notices a close to 55% diminishing of the areas with very-high-density vegetation, that is from around 87,000 hectares by 1990 to around 40,000 hectares by 2011. A higher threshold value (0.57) preserves a 50%-plus difference between the two years, that is from around 63,700 hectares by 1990 to around 30,000 hectares by 2011.

### Clear-cutting of forest ecosystems

**Figure 7:**
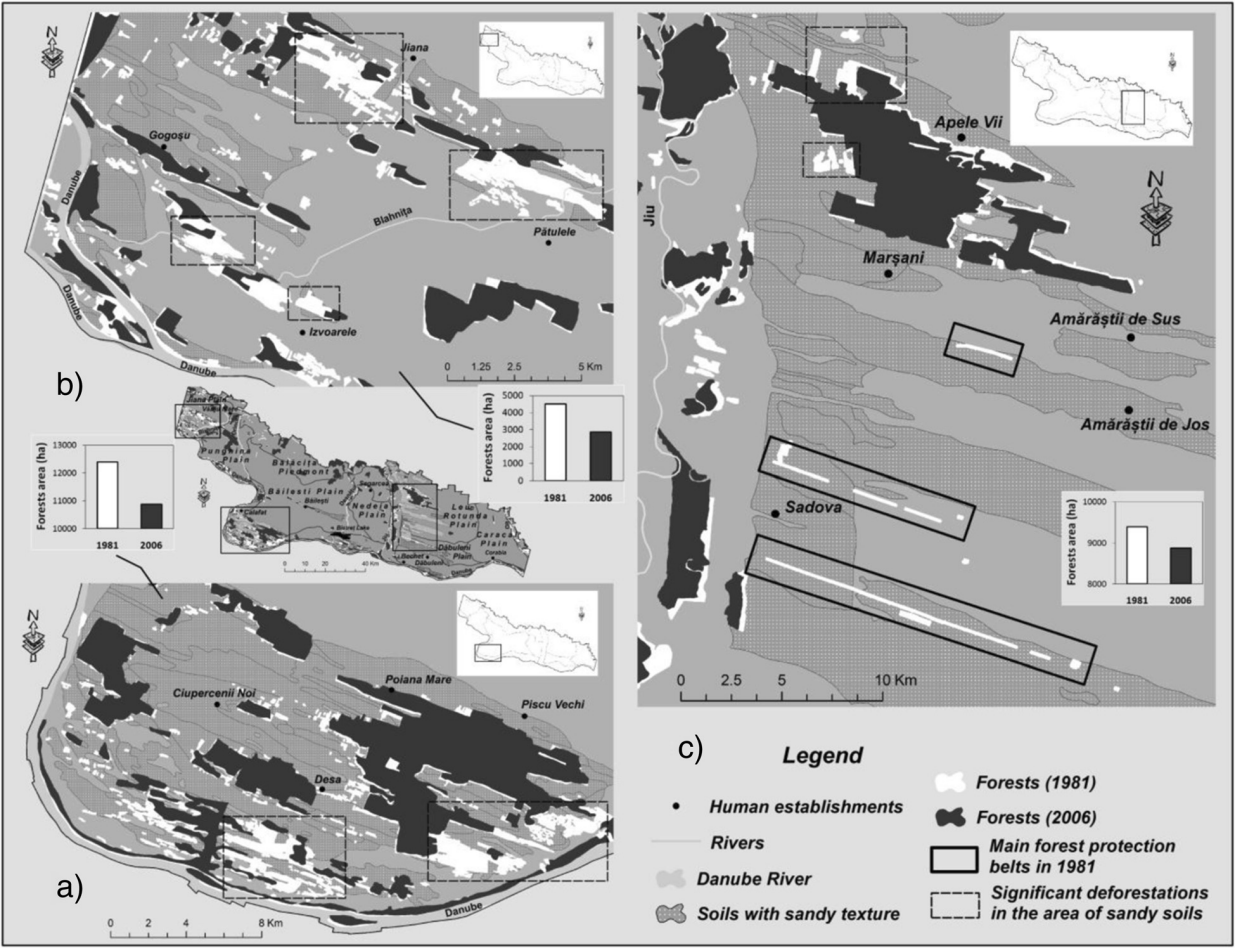
Evolution of the forest areas in the Ciupercenii Noi-Rast (a), Jiana–Patulele– Izvoare (b) and Dabuleni–Apele Vii (c) areas during 1981–2006.

The analysis of the evolution of forest-covered areas across the overall study area highlights a 7.2% decline of the respective areas (4,485 hectares), that is, from 62,058 hectares by 1981 to 57,573 hectares by 2006. However, this process registers certain quite sizeable fluctuations in space. For a more detailed assessment of the situation, three case studies were selected, located at the same time in the areas with the steepest drops in forest areas and on sandy soil: Ciupercenii Noi - Rast (in the Southern part of the study area), Jiana – Patulele – Izvoare (the Western part) and Dabuleni – Apele Vii (the Eastern part) (Figure 7).

*Case study: Ciupercenii Noi – Rast.* This case stands out because of a rapid change of forest areas, which shrank by 12.3% (1,515 hectares) during the 25-year-span, that is from around 12,397 hectares by 1981 to 10,881 hectares by 2006 (Figure 7a). The most visible decline of the forest areas occurred to the South and the South-East of the township of Desa in an area of sand deposits, with false-acacia (*Robinia pseudoacacia*) the predominant species, as well as in the Danube river valley, with white willow (*Salix alba*) and poplar (*Canadian poplar* in general) the predominant species.

The Markov matrix method was used to achieve a detailed analysis of the evolution of the forest ecosystems in that area (Table 1). The area analyzed was divided into 8 main categories of land use, and forest areas were divided according to the three dominant forest species of the area analyzed: false-acacia, poplar and willow.

**Table 1:** Changes in land use (1981 – 2008) as shown by the Markov matrix method in the Ciupercenii Noi – Rast area (ha)

**Land use type/Forest species**	**Bodies of water**	**Agricultural**	**Built areas**	**Marshes**	**Sandy areas**	**Poplar**	**False-acacia**	**Willow**	**Total 2008**
Bodies of water	11.9	1.9	0.1	0.7	-	0.3	-	-	14.9
Agricultural	166.9	20995.7	55	1747.5	76.3	1030.4	2275.2	6.8	26353.8
Build areas	57	481.3	1665.3	3.8	-	3.9	13.3	-	2224.6
Marshes	116.7	500.4	-	1448.4	0.2	14.8	8	-	2088.5
Sandy areas	0.9	204	-	5	72.6	17.7	9.5	-	309.7
Poplar	4.1	191.1	-	13.1	0.8	1720	28.4	-	1957.5
False-acacia	0.3	897.1	12.4	13	2.1	95	6566.7	-	7586.6
Willow	-	6.2	-	-	-	0.3	49.7	83.6	139.8
Total 1981	357.8	23277.7	1732.8	3231.5	152	2882.4	8950.8	90.4	40675.4

Evaluation of the matrix is done both on the vertical and on the horizontal. The data in the land-type categories, listed in columns (on the vertical) represent surface losses to the detriment of the data on the horizontal. The gains are listed in rows (on the horizontal), meaning that the difference between losses and gains is the positive or negative balance of the areas of the types of land demarcated by the two reference years. The figures in the column-row intersections of a single category of land mean areas unmodified during the time span analyzed.

Out of the total area covered with false-acacia, most of it (1,378 hectares, the difference between losses and gains) was eliminated especially to the detriment of agricultural land (grazing land, meadows, vineyards, orchards, arable land). The situation is relevant at least up to 1990 when a large part of the forest-covered land was deforested so as to expand agricultural land.

*Case study: Jiana – Patulele.* In the Jiana – Patulele area (Figure 7b) one notices the 37% decline in forest surface (1,666 hectares) during 1981–2006. In this case, too, the disappearance of the forest ecosystems occurred mostly to the detriment of sandy soil, thus bringing about the onset of wind erosion, with negative effects on the environment and human settlements.

In order to create a Markov matrix, three dominant forest species present in the study area were taken into consideration: false acacia, oak and poplar (Table 2). In this case, too, the loss in forested areas occurred at the expense of acacia-covered areas. Out of the 222 hectares lost (total losses – gains balance), close to 100% occurred in favor of the agricultural land. Poplar registered 20% losses (90 hectares) from 1981 to 2008, while oak registered a slight 3% increase in area (22 hectares).

**Table 2:** Changes in land use (1981 – 2008) as shown by the Markov matrix method in the Jiana – Pătulele area (ha)

**Land use type/Forest species**	**Bodies of water**	**Agricultural**	**Build areas**	**Marshes**	**Sandy areas**	**Poplar**	**False-acacia**	**Oak**	**Total 2008**
Bodies of water	1.2	0.2	-	-	-	-	-	-	1.4
Agricultural	28	23819.8	87.4	3646.3	61.4	137.7	747.5	42	28570.1
Build areas	-	152.3	1227.2	5.2	-	-	1.1	-	1385.8
Marshes	0.3	122.1	0.5	705.9	-	1.4	0.5	0.5	831.2
Sandy areas	-	112.5	-	5.7	9.3	8.2	-	-	135.7
Poplar	-	52.4	-	5.2	-	336.3	0.4	-	394.3
False-acacia	7.7	469.6	0.9	49.3	-	0.1	2825.5	0.3	3353.4
Oak	5.4	44.5	-	14.4	-	-	0.2	664.6	729.1
Total 1981	42.6	24773.4	1316	4432	70.7	483.7	3575.2	707.4	35401

**Figure 8:**
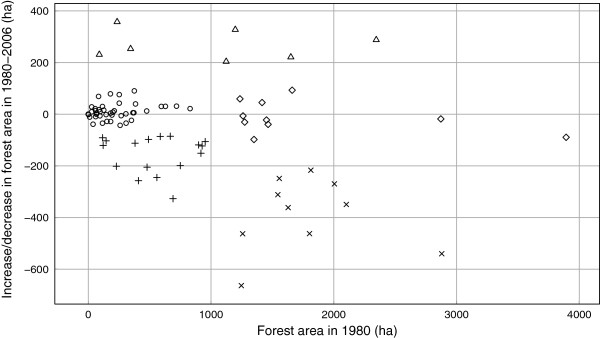
Commune clusters grouped in terms of the size and evolution of forested areas.

**Figure 9:**
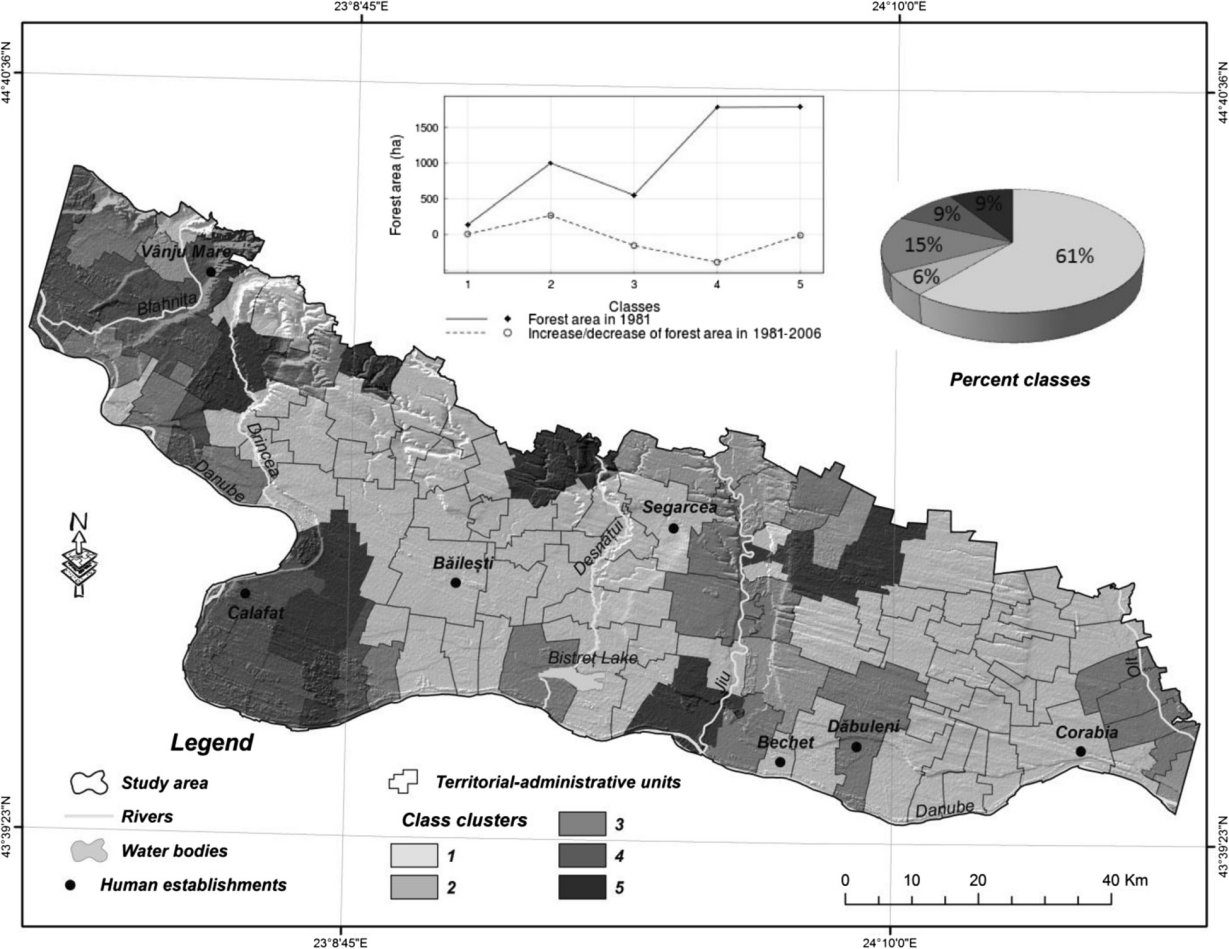
Analysis of the evolution of forest ecosystems at the level of administrative-territorial units.

*Cluster analysis at commune (territorial-administrative units) level.* The cluster analysis revealed 5 groups (clusters) of communes (Figure 8). Out of the total number of communes in the study area, the 7 communes, which constitutes the class 2 group on the figure, are the only ones to display a visible trend towards an increase of forest-covered areas, and they account for a mere 6% of the area’s surface. The class-1 and class-5 communes are characterized by relatively limited changes in forest-covered surfaces, irrespective of their size. The communes in classes 3 and 4 stand out because of the visible trend towards a decrease of forest surfaces. Here, the communes with the biggest surfaces register the biggest drops, as well (class 4). However, the communes in class 3, despite the relatively small drops in forest-covered areas, are those that suffer the worst because of deforestation, because the respective forests are small. In all, classes 3 and 4 account for circa 1/4 of the total surface of the study area. The spatial distribution of the classes reveals that communes in the South-Western and Western parts of the region, as well as those along the Jiu river valley, are worst hit by the decrease of forested areas (Figure 9).

## Discussions

Transformation of the local climate is one of the major causes of the escalation of aridization. The analysis of the De Martonne (Figure 2) and UNEP (Figure 3) aridity indexes reveals the obvious trend towards aridization in the past three decades. Therefore, as shown by the evolution of both indicators, we may conclude that, from the mid-1980s onwards, a climate with semiarid features sets in the study area, with a visible trend towards an escalation of aridization. The amplifying of aridity conditions had a significant impact on forest ecosystems degradation. Taking the example of 1992 and 2000, when the Martonne index presented the lowest values (the minimum being 11.7 mm/°C, respectively 11.9 mm/°C, registered at Craiova weather station, values corresponding to semiarid climatic conditions) (Figure 2). Apparently, they were characterized by very low amounts of annual rainfall (254.8 mm and 271.2 mm) and a high thermal stress annually (average annual temperature of 11.8°C, 12.7°C respectively). Given the contribution of very low moisture and temperature conditions (which led to heat stress with surpassing 11.5°C, generally considered critical, which can lead to dryness species of acacia), it can be concluded that all these climatic conditions have considerably reduced the ecologic potential of the forest ecosystems, causing their degradation over the past two - three decades.

### Forest ecosystem degradation

Climate change impact on forest ecosystems is revealed by the two indexes, NDVI and MSAVI2. As NDVI shows, extending of critical areas – lacking vegetation cover (NDVI < 0.1) – by 85 thou. ha in 2011 comparing to 1990 has taken place at the expenses of forest areas. This process was mainly caused by the increase in the frequency of droughts starting 1980 [33], events that caused the drying-up of forest species on significant surfaces. Similar trends are shown by MSAVI2 index, however, increase in critical areas is less spectacular (15 thou. ha).

There is a strong relationship between the changes of the two indexes. Although by 1990 there was no obvious spatial correlation between the exposed areas as shown by the two indexes as a result of the qualitatively speaking better situation of the vegetal layer (a situation caused by the more favorable ecologic potential in the context of a less intense aridization of the area), by 2011 there can be noticed a much more marked spatial correlation (Figure 5). The existence of a 0.94 R^2^ determination quotient, as well as the existence of the 0.97 r correlation quotient, emphasizes a close spatial correlation of the no-vegetation areas as shown by means of the two indexes, NDVI and MSAVI2. Thus, the close mathematical correlation present at the level of the two indexes suggests a bilateral spatial evolution of degradation of the vegetal layer and implicitly of the forest ecosystems, as a result of the increase in the intensity of aridization in the past two decades.

Analysis of forest areas, which corresponds to higher thresholds of the normalized digital vegetation index (NDVI > 0.55, >0.57) reveals significant reduction in 2011 in comparison to 1990 (Figure 6). However, these significant drops that occur at high thresholds, assigned to forest areas, do not reflect the disappearance of 50% of the forests from 1990 to 2011, but merely a decline in the spectral brightness of the vegetation. Thus, while by 1990, one notices on the pixel histogram a high concentration of the values above the 0.55 and 0.57 thresholds (Figure 6), values in general typical of forested areas, by 2011 one notices a significant drop in those values above the respective thresholds. This decline occurred to the detriment of the increase of the concentration of values approximately located in the 0.15 - 0.5 range. One thus notices a decline in the spectral brightness of the very dense vegetation in the context of the degradation of forest ecosystems.

### Clear-cutting of forest ecosystems

*Case study: Ciupercenii Noi – Rast.* Starting with the politico-economical transition begun in early 1990 and up to the present, a part of the agricultural land was abandoned as a result of its return to the former owners and the owners’ subsequent neglect. Thus, although by 2008 the farmland surface was circa 3,076 hectares bigger than by 1981, as a result of expansion, mainly to the detriment of false-acacia forests, nowadays a large part of the agricultural land is unused. However, one can also notice, by 2008, expansion of the false-acacia areas (because of reforestation, natural regeneration) to the detriment of other land types, so that the final balance from 1981 to 2008 is 1,364 hectares lost.

The situation is similar in the case of poplar, with 90% of the decline of the poplar surface during 1981 – 2008 (839 hectares) replaced by agricultural land, out of a total 925 hectares lost (Table 1). The areas covered with willow species registered a positive evolution, expanding by 65% (50 hectares) over the 27-year time span. One of the main reasons is connected to their location in the Danube river valley, an area with minimal anthropogenic influence, which allowed their natural regeneration.

Other causes of the decline/deforestation of false-acacia and poplar areas are linked to these species drying up on significant areas, as a result of both changes in the climate (the increase of drought frequency), and changes in local conditions (changes in the local hydrological regime as a result of the decline in groundwater levels, as a result of the water withdrawing from the dead arms of the Danube). In the context of the drying up of forest species, excessive forest-cleaning work, in terms of the local Calafat and Poiana Mare forestry districts’ cutting dead trees is another important cause for the shrinking of forest areas [19,20]. At the same time, the surplus end-balance between the pace of logging and the pace of afforestation is another cause for the decline of forest-ecosystem areas [20].

*Case study: Jiana – Patulele.* The forest ecosystem (false-acacia and poplar) loss surfaces occurred in general in favor of agricultural land (a process eased by Law 18/1990), and the explanations are similar to those mentioned in the Ciupercenii Noi – Rast case.

Other causes are related to deforestation carried out inside the forestry districts in the region (Jiana, Simian) as a result of logging outpacing forestation work. In particular, the surplus balance of forest-cleaning actions (cutting down, on large areas, more than the planned number of dead acacias in the context of long droughts) is a major cause of the diminution of the forest ecosystems on large surfaces [21]. There are also other related causes, too, such as the absence of the mechanized tools to prepare the land, insufficient afforestation materiel, low financial resources, increasingly limited manpower, among others, which outweigh the afforestation capacity of forestry districts [21]. At the same time, the modification of the water flow balance upon the construction of the Portile de Fier II hydro power plant, upstream on the Danube river, is yet another cause for the drying up (and later excessive cleaning) of *Canadian poplar* species present in the areas between the sand dunes [21].

*Case study: Dabuleni – Apele Vii.* The last case study, Dabuleni – Apele Vii (Figure 7c) is a special situation because of the manner of technical administration of the forest ecosystems in the region. The construction of the Sadova - Corabia irrigation system during 1972–1974 involved the deforestation of around 5,000 hectares of forest [18] during 1969–1970, a situation that made it necessary to create a vast system of shelter belts as a result of the environment imbalances generated by the reactivation of the sand dunes. As the acacia shelter belts present up to 1970 in the form of copses of trees were cut down to allow the construction of the irrigation system, and later on reconverted for agriculture, most of the Dabuleni – Apele Vii study area has been arranged so far into acacia shelter belts, with protective purposes. Initially, these belts covered an area of around 1,600 hectares across a total distance of 1,700 km, spanning an area of sandy soil of around 45,000 hectares [26].

In general, the distance between the belts is limited, and they are located at close distance (288 m) so as to limit the effects of wind erosion, protect crops against physical damage caused by sand-storms, and limit the silting of water installations, among others. Although the average width is 8–10 meters, and their predominant direction is Northeast – Southwest, perpendicular to the direction of the predominant winds in the region, the spatiotemporal analysis (Figure 7c) only captured a part of the shelter belts (the densest), as a result of the limited spatial resolution of the Corine Land Cover, 2006 database. Therefore, in the context of the presence of the spatial distribution of the forest vegetation in the form of shelter belts rather than copses of trees, the use of the Markov matrix method was no longer viable.

However, mention must be made of several negative aspects concerning the present state of the shelter belts. Quantitatively speaking, a large part of them have disappeared as a result of drying up in the context of severe aridization, but especially because of the numerous instances of illegal tree-cutting, in particular after 1990 [30]. Qualitatively, shelter belts are in an advanced state of deterioration, and the causes are connected both to the deficient administration at the level of forestry districts (the absence of the proper technologies to restore shelter belts, the absence of security that would fight illegal tree-cutting and the fragmentation of shelter belts, among others), as well as to the changes in the ecologic potential in the context of the modification of the weather conditions [17-21].

## Conclusions

Climate aridization in Southern Oltenia is a real process that has been on a more or less constant trend in the past half-century. Arid climate conditions set in from the mid-1980s on in the study area, while the aridization process goes on; this situation led to the escalation of the climate stress on the forests. The aridization of the weather conditions, the increase in climate stress leads to the premature drying-up of the vegetation in the area, the degradation of the forest ecosystems. The results of this study, based on remote-detection materials, spanning the past 20 years, confirm the presence and the severity of this process, which eventually leads to the decline in the quality of the ecosystem services offered by forests.

Aridization, a process that occurs independently from local factors, is accompanied by deforestation. Deforestation began in the second half of the 20^th^ century and it has continued, intermittently, up to the present time. Curiously, neither the 1990 events nor Law 18 did anything to introduce a new trend in the evolution of forested areas in the region, but, on the contrary, they boosted the previous trend and diversified it in space. Our results, based on comparing forest surfaces over the past 30 years, reveal an overall 7% decline of forest surface in the area. At regional scale, the figure rises as high as 33%, which justifies our view of a large-scale deforestation process. Isolated attempts at afforestation on a significant scale (in the case of 7 communes) seem imperceptible in the context of the entire region.

The case studies emphasize the fact that the areas most vulnerable to aridization (sandy-texture soil and sand dunes) are the first to suffer deforestation on a massive scale. The cross-species analysis indicates that false acacia (*R. pseudoacacia),* a species frequently used in the past to stabilize frail soil, suffers the worst as a result of deforestation. Surface losses, but smaller in scale, also plagued poplars (*Populus sp.*), another species used in land improvement (especially Canadian poplar – *P. canadensis).*

In the past few years, several regulations were drafted at governmental level, whose purpose was alleviating the imbalances visible at territorial system level. According to those regulations, the administrative units with inadequate surface areas are required to take steps so as to expand forest areas, by means of actions that would encourage land owners to change the destination of the land (Government decree 994/2004; Law 46/2008 – Forestry Code).

Alleviating the imbalances identified is conditioned by the implementation of territorial management strategies, which would offer to the decision-makers efficient solutions in terms of the decision-making component of the territorial systems impacted [61-66].

As the results of our research indicate in the present study, forests in Southern Oltenia have suffered a lot in the past 30 years, in terms of both the decline of the quality of forest ecosystems and the absolute surface they cover. Here, the quasi-natural global causes (climate change) and local anthropogenic causes (logging) acted unidirectionally towards the destruction of the forested areas in the study areas. If measures to improve the situation are not urgently taken, the continuation of the current trend will mean the consequences in natural and socio-economic terms would be very grave.

## Abbreviations

ECAD: European climate assessment dataset; PET: Potential evapotranspiration; I ar. DM: De Martonne aridity index; NIR: Near infrared spectral band; RED: Red spectral band of a multispectral satellite image; NDVI: Normalized difference vegetation index.

## Competing interests

The authors declare that they have no competing interests.

## Authors’ contributions

RP participated in the analysis and interpretation of data, involved in drafting the manuscript or revising; IS participated in the conception of the study and performed the statistical analysis; DP participated in the conception, or acquisition of data, or analysis and interpretation of data, participated in the given final approval of the version to be published. All authors read and approved the final manuscript.

## References

[B1] Fuhrer J Beniston M Fischlin A Frei C Goyette S Jasper K Pfister C Climate risks and their impact on agriculture and forests in Switzerland Clim Change 2006 79 79 102

[B2] Kirilenko AP Sedjo RA Climate change impacts on forestry Proc Natl Acad Sci USA 2007 104 19697 19702 18077403 10.1073/pnas.0701424104 PMC2148360

[B3] Chhin S Hogg EH Lieffers VJ Shongming H Potential effects of climate change on the growth of lodgepole pine across diameter size classes and ecological regions For Ecol Manage 2008 256 1692 1703

[B4] Gimmi U Wohlgemuth T Rigling A Hoffmann C Burgi M Land-use and climate change effects in forest compositional trajectories in a dry central-alpine valley Ann For Sci 2010 67 701

[B5] Veron SR Paruelo JM Desertification alters the response of vegetation to changes in precipitation J Appl Ecol 2010 47 1233 1241

[B6] Epron D Dreyer E Long-term effects of drought on photosynthesis of adult oak trees [Quercuspetraea (Matt.) Liebl. and Quercusrobur L.] in a natural stand New Phytol 1993 125 381 389 10.1111/j.1469-8137.1993.tb03890.x 33874494

[B7] Roupsard O Gross P Dreyer E Limitation of photosynthetic activity by CO₂ availability in the chloroplasts of oak leaves from different species and during drought Ann Sci For 1996 53 243 254

[B8] Scotti I Adaptive potential in forest tree populations: what is it, and how can we measure it? Ann For Sci 2010 67 801

[B9] Chmura DJ Anderson PD Howe GT Harrington CA Halofsky JE Peterson DL Shawe DL St Clair JB Forest responses to climate change in the northwestern United States: ecophysiological foundations for adaptive management For Ecol Manage 2011 261 1121 1142

[B10] IPCC Climate change 2007: the physical science basis. Contribution of working group in to the fourth assessment report to the intergovernmental panel on climate change 2007 Cambridge: Cambridge University Press

[B11] Paltineanu C Mihailescu IF Prefac Z Dragota CS Vasenciuc F Nicola C Combining the standardised precipitation index and climatic water deficit in characterising droughts: a case study in Romania Theor Appl Climatol 2009 97 219 233

[B12] Stringer CL Scrieciu S Reed SM Biodiversity, land degradation, and climate change: participatory planning in Romania Appl Geogr 2009 29 77 90

[B13] Blujdea V Foresters’ perception concerning the impact of climate changes on forests Annals of Forest Research 2005 48 1 151 160

[B14] Sasaki N Putz FE Critical need for new definitions of “forest” and “forest degradation” in global climate change agreements Conserv Lett 2009 2 226 232

[B15] Baskent EZ Kadiogullari AI Spatial and temporal dynamics of land use pattern in Turkey: a case study in Inegol Landsc Urban Plan 2007 81 316 327

[B16] WorldClim - global climate datahttp://www.worldclim.org

[B17] Forest Research and Management Institute Jiana forestry-planning document 1997 Voluntari: General Study

[B18] Forest Research and Management Institute Sadova forestry-planning document 2003 Voluntari: General Study

[B19] Forest Research and Management Institute Calafat forestry-planning document. General study 2004 Voluntari: General Study

[B20] Forest Research and Management Institute Poiana Mare forestry-planning document 2004 Voluntari: General Study

[B21] Forest Research and Management Institute Simian forestry-planning document 2010 Voluntari: General Study

[B22] Nuta S Posea G, Bogdan O, Zăvoianu I, Buza M, Bălteanu D, Niculescu G Câmpia olteniei. Relieful Geografia româniei, volumul V 2005 Bucharest: Romanian Academy Publishing House 143 150

[B23] Pedology and Agrochemistry Research Institute The soils map in electronic format, 1:200,000 2013 Bucuresti http://www.icpa.ro/

[B24] Patroescu M Posea G, Bogdan O, Zăvoianu I, Buza M, Bălteanu D, Niculescu G Câmpia olteniei. Vegetatia si fauna Geografia româniei, volumul V 2005 Bucharest: Romanian Academy Publishing House 154 156

[B25] Dumitrascu M Modificari ale peisajului în câmpia olteniei 2006 Bucuresti: Academiei Române

[B26] Nuta S Caracteristici structurale si functionale ale perdelelor forestiere de protectie a câmpului agricol din sudul Olteniei Analele ICAS 2005 48 16 169

[B27] Traci C Impadurirea terenurilor degradate 1985 Bucharest: Ceres Publishing House

[B28] Constandache C Nistor S Ivan V Impadurirea terenurilor degradate ineficiente pentru agricultura din sud-estul tarii Analele ICAS 2006 49 187 204

[B29] Dumitrascu M Modificari ale peisajului în câmpia olteniei 2006 Bucharest: Romanian Academy Publishing House

[B30] Achim E Manea G Vijulie I Cocos O Tirla L Ecological reconstruction of the plain areas prone to climate aridity through forest protection belts. Case study: dabuleni town, oltenia plain, Romania Procedia Environmental Sciences 2012 14 154 163

[B31] Marinica I Vaduva I Comparison between the oltenia plain and the southern dobrogea plateau in terms of pluviometric deficit, forum geografic Studii şi cercetari de geografie şi protecţia mediului 2010 9 57 64

[B32] Vladut A Ecoclimatic indexes within the oltenia plain, forum geografic Studii si cercetari de geografie si protectia mediului 2010 9 49 56

[B33] Dragota CS Dumitraşcu M Grigorescu I Kucsicsa G The climatic water deficit in south Oltenia using the Thornthwaite method, forum geografic Studii şi cercetari de geografie şi protecţia mediului 2011 10 140 148

[B34] Pravalie R Climate issues on aridity trends of southern oltenia in the last five decades Geographia Technica 2013 1 70 79

[B35] Prăvălie R Peptenatu D Sîrodoev I The impact of climate change on the dynamics of agricultural systems in south-western Romania Carpathian Journal of Earth and Environmental Sciences 2013 8 3 175 186

[B36] Peptenatu D Sîrodoev I Pravalie R Quantification of the aridity process in south-western Romania Journal of Environmental Health Science & Engineering 2013 11 3 24499565 10.1186/2052-336X-11-5 PMC3776297

[B37] Ignat P Gherghina A Vrînceanu A Anghel A Assessment of degradation processes and limitative factors concerning the areno sols from dabuleni – Romania, forum geografic Studii şi cercetari de geografie şi protecţia mediului 2009 8 64 71

[B38] European Climate Assessment & Dataset European climate assessment & dataset[http://eca.knmi.nl/dailydata/customquery.php]

[B39] De Martonne E Une nouvelle fonction climatologique: L’indice d’aridite La Meteorologie 1926 2 449 458

[B40] UNEP World atlas of desertification 1992 London, UK: Edward Arnold

[B41] Thornthwaite CW An approach toward a rational classification of climate The Geographical Rev 1948 38 1 55 94

[B42] Paltineanu C Mihailescu IF Seceleanu I Dragota CS Vasenciuc F Using aridity indexes to describe some climate and soil features in eastern Europe: a Romanian case study Theor Appl Climatol 2007 90 263 274

[B43] Maselli F Monitoring forest conditions in a protected Mediterranean coastal area by the analysis of multiyear NDVI data Remote Sens Environ 2004 89 423 433

[B44] NASA Landsat Program Landsat Program (1990). Landsat TM scenes L5184029_02919900711 and L5184030_03019900711, L1T 1990 Sioux Falls: USGS

[B45] NASA Landsat Program Landsat TM scenes L5184029_02920110822 and L5184030_03020110822, L1T 2011 Sioux Falls: USGS

[B46] Chander G Markham BL Barsi JA Revised landsat-5 thematic mapper radiometric calibration IEEE Geosci Remote Sens Lett 2007 4 490 494

[B47] Landsat Mission [http://landsat.usgs.gov/science_calibration.php]

[B48] Helldén U Tottrup C Regional desertification: a global synthesis Global Planet Change 2008 64 169 176

[B49] Barbosa HA Huete AR Baethg WE A 20-year study of NDVI variability over the northeast region of brazil J Arid Environ 2006 67 288 307

[B50] Xu D Kang X Qiu D Zhuang D Pan J Quantitative assessment of desertification using landsat data on a regional scale – a case study in the Ordos plateau, china Sensors 2009 9 1738 1753 22573984 10.3390/s90301738 PMC3345839

[B51] Sternberg T Tsolmon R Middleton N Thomas D Tracking desertification on the Mongolian steppe through NDVI and field-survey data International Journal of Digital Earth 2011 1 50 64

[B52] Huete AR A soil-adjusted vegetation index (SAVI) Remote Sens Environ 1988 25 295 309

[B53] Qi J Chehbouni A Huete AR Kerr YH Modified soil adjusted vegetation index (MSAVI) Remote Sens Environ 1994 48 119 126

[B54] Van der Meer F Bakker W Scholte K Skidmore A Jong de S Clevers J Addink E Epema G Spatial scale variations in vegetation indices and above-ground biomass estimates: implications for MERIS International Journal Remote Sensing 2001 17 3381 3396

[B55] Boles SH Xiao XM Liu JY Zhang QY Sharav M Chen SQ Land cover characterization of temperate east Asia using multi-temporal vegetation sensor data Remote Sens Environ 2004 90 477 489

[B56] Baugh WM Groeneveld DP Broadband vegetation index performance evaluated for a low-cover environment Int J Remote Sens 2006 21 4715 4730

[B57] Liu ZY Huang JF Xin HW Dong YP Comparison of vegetation indices and red-edge parameters for estimating grassland cover from canopy reflectance data J Integr Plant Biol 2007 49 299 306

[B58] Petit T Scudder T Lambin E Quantifying processes of land-cover change by remote sensing: resettlement and rapid land-cover changes in south-eastern Zambia Int. J. Remote Sensing 2001 17 3435 3456

[B59] Rimal B Urban growth and land use/land cover change of Biratnagar sub-metropolitan city, Nepal Appl Rem Sens J 2011 2 1 6 15

[B60] NASA earth observatory http://earthobservatory.nasa.gov/Features/MeasuringVegetation/

[B61] Ianos I Peptenatu D Pintilii RD Draghici C About sustainable development of the territorial emergent structures from the metropolitan area of Bucharest Environmental Engineering And Management Journal 2012 11 9 1535 1545

[B62] Braghina C Peptenatu D Constantinescu Ş Pintilii RD Draghici C The pressure exerted on the natural environment in the open pit exploitation areas in Oltenia Carpathian Journal of Earth and Environmental Sciences 2010 5 33 40

[B63] Braghina C Peptenatu D Draghici C Pintilii RD Schvab A Territorial management within the systems affected by mining: case study the south-western development region in Romania Iran 2011 8 342 352

[B64] Caliskan E Environmental impacts of forest road construction on mountainous terrain Iran 2013 10 23 10.1186/1735-2746-10-23 PMC3627898 23497078

[B65] Peptenatu D Merciu C Merciu G Draghici C Cercleux LA Specific features of environment risk management in emerging territorial structures Carpathian Journal of Earth and Environmental Sciences 2012 7 135 143

[B66] Peptenatu D Pintilii RD Draghici C Stoian D Environmental pollution in functionally restructured urban areas: case study – the city of Bucharest Iran 2010 7 87 96

